# Advanced Imaging Tools Rather Than Hemodynamics Should Be the Primary Approach for Diagnosing, Following, and Managing Pulmonary Arterial Hypertension

**DOI:** 10.1016/j.cjca.2015.01.019

**Published:** 2015-04

**Authors:** Mario Gerges, Christian Gerges, Irene M. Lang

**Affiliations:** Division of Cardiology, Department of Internal Medicine II, Vienna General Hospital, Medical University of Vienna, Vienna, Austria

## Abstract

Pulmonary hypertension (PH) is currently defined based on invasive measurements: a resting pulmonary artery pressure ≥ 25 mm Hg. For pulmonary arterial hypertension, a pulmonary arterial wedge pressure ≤ 15 mm Hg and pulmonary vascular resistance > 3 Wood units are also required. Thus, right heart catheterization is inevitable at present. However, the diagnosis, follow-up, and management of PH by noninvasive techniques is progressing. Significant advances have been achieved in the imaging of pulmonary vascular disease and the right ventricle. We review the current sensitivities and specificities of noninvasive imaging of PH and discuss its role and future potential to replace hemodynamics as the primary approach to screening, diagnosing, and following/managing PH.

## Résumé

L’hypertension pulmonaire (HP) est actuellement définie selon des méthodes effractives de mesure : une pression artérielle pulmonaire ≥ 25 mm Hg au repos. Pour définir l’hypertension artérielle pulmonaire, une pression artérielle pulmonaire d’occlusion ≤ 15 mm Hg et une résistance vasculaire pulmonaire > 3 unités Wood sont également requises. Par conséquent, le cathétérisme cardiaque droit est inévitable pour le moment. Malgré cela, le diagnostic, le suivi et la prise en charge de l’HP par des techniques non effractives évoluent. L’imagerie des maladies vasculaires pulmonaires et du ventricule droit a connu d’importants progrès. Nous passons en revue la sensibilité et la spécificité actuelles de l’imagerie non effractive de l’HP, et discutons de ses rôles et de son potentiel futur de remplacement de l’hémodynamique comme principale approche en matière de dépistage, de diagnostic, de prise en charge/suivi de l’HP.

Pulmonary arterial hypertension (PAH) is an orphan condition with high morbidity and mortality. Despite increased awareness of pulmonary hypertension (PH), data indicate that the majority of patients are still diagnosed in late stages of the disease. A higher World Health Organization functional class is associated with poorer median survival, illustrating the importance of early diagnosis. In this article, we were asked to defend the value of noninvasive imaging in the diagnosis and follow-up of PH. Although we agree that at this point, invasive assessment remains essential, in the long term it is hoped that noninvasive methods will eliminate the need for invasive assessment. Our original mandate was to discuss PAH; however, because this is a rare condition with relatively little information available, we have broadened our approach to include PH in general.

## Limitations of Invasive Assessment

### Invasive hemodynamic assessment by right heart catheterization is relatively safe but has technical limitations

At the Nice 5th World Symposium on PH, right heart catheterization (RHC) was confirmed as essential for the diagnostic workup of PH to assess the severity of the disease and to perform a vasoreactivity test.^1-3^ However, RHC is associated with rare, albeit serious, procedure-related complications, including death. In an analysis of 7218 RHC procedures performed in experienced PH centres, 76 serious adverse events, including 4 fatalities, were observed. The most common serious adverse events were supraventricular and ventricular tachycardia, vagal reactions, and systemic hypotension.^4^ Although RHC is relatively safe, reports of complications do appear, even in expert centres.^5^

Data acquisition during RHC requires resting supine patients. There is no standard operating procedure for capturing hemodynamic changes that occur with an upright posture or with physical activity using RHC. In addition, hemodynamic measurements acquired by RHC are subject to intraindividual spontaneous variability and represent only a hemodynamic snapshot.^1,6^

Routine RHC relies on the use of fluid-filled catheters, which have an insufficient frequency response.^7^ Standard Swan-Ganz catheter manometry systems used in clinical practice have a frequency response of 12 Hz, whereas a minimum of 50 Hz would be required for the assessment of instantaneous pressure signals.^7^ Fluid-filled catheters require fast flushes to remove air bubbles in the monitoring system, which account for most of the variability compared with the true gold-standard high-fidelity micromanometer-tipped catheters.^7^ In contrast to high-fidelity micromanometer-tipped catheter systems, fluid-filled catheter transducers have to be positioned at a “zero reference level,” which is most accurately obtained at midthoracic level or at one third of the thoracic diameter below the anterior thorax surface.^8^ A deviation of 1 cm of the transducer from zero level affects pressures by 0.78 mm Hg, thus leading to significantly different results if 2 different zero reference levels are used in a single patient.^8^

### Currently used invasive cardiac output measurements estimate but do not measure true cardiac output

The gold standard for the assessment of cardiac output (CO) is the direct Fick method in which CO equals O_2_ consumption divided by the difference between arterial and venous O_2_ content. Although O_2_ consumption can be measured accurately, that measurement is cumbersome, and many laboratories use standard tables for an assumed value instead of direct measurements. Such estimation may cause an error of as much as 40% in the assessment of CO.^9^ Most laboratories now use thermodilution based on an indicator dilution methodology to measure CO.^10^ When compared with the direct Fick method, thermodilution measurements show little bias, with a mean difference of 0.1 L/min and a confidence interval of 0.2 L/min, corresponding to excellent accuracy even in the presence of tricuspid regurgitation, but limits of agreement are ± 1 L/min, corresponding to moderate precision.^11^

### Need for an integrated diagnostic approach

Clinically significant information is gained from RHC that helps guide decisions. A restrictive use of RHC may delay a timely diagnosis and treatment.^6^ Still, the simple distinction between pre- and postcapillary PH is a task that often cannot even be achieved by invasive RHC. In particular, heart failure with preserved ejection fraction is commonly misdiagnosed as precapillary PH.^12-14^ Unresolved issues are the assessment of precatheterization fluid status, standardization of fluid loading,^3,15,16^ and mean pulmonary arterial wedge pressure measurements—end-expiratory or as pressure-time integral.^16,17^ The interpretation of invasive hemodynamics is meaningless outside the context of the clinical picture, in particular echocardiography.^1,3^ To manage the growing number of PH cases resulting from left heart disease (group 2 PH) and caused by lung disease/hypoxia (group 3 PH) in the general population, successful noninvasive diagnostic algorithms combining multiple parameters have been developed to avoid unnecessary RHC.^1,18^

## Present Value of Noninvasive Techniques

### Advanced imaging tools are useful for screening

Transthoracic Doppler echocardiography is the predominant screening modality in early stages of diagnosis to assess right ventricular (RV) structure and function, including the degree of ventricular remodelling as well as the derivation of RV systolic and diastolic pressures and analysis of contraction timing,^19-23^ thus providing a reliable method for the early detection of PH, with a particularly high sensitivity and specificity in systemic sclerosis (Table 1). Recently, software programs for 2-dimensional (2D) strain analysis by speckle tracking have been applied to evaluate the right ventricle.^31^ Furthermore, significant progress has been made in the use of knowledge-based reconstruction of 3D RV structure and function from 2D images.^32^ Studies have suggested that 3D echocardiographic imaging of the right ventricle is feasible, and its results compare well with magnetic resonance imaging (MRI).^33,34^

Theoretically, imaging of the pulmonary vasculature should be more sensitive to screening because this is where disease starts; yet, the available methods do not appear to have reached adequate sensitivity and specificity for that purpose.^35^

### Advanced imaging tools are useful for diagnosis

Any patient with unexplained PH should be evaluated for chronic thromboembolic PH (CTEPH). Diagnostic algorithms for PH include ventilation/perfusion (V/Q) scintigraphy,^1,36-40^ multidetector computed tomography (CT), and cardiac MRI (cMRI).^41^ Although a mosaic pattern is common in CTEPH, it occurs in up to 12% of patients with PAH.^42^ MRI of the pulmonary vasculature is still considered inferior to CT but may be preferred according to local practice.^43^ Recent advances—such as dual-energy CT,^44^ cone-beam CT, electrocardiographic gated 320-row area detector CT, and lung perfusion MRI—are about to change paradigms in pulmonary vascular imaging. In a pilot study, dynamic contrast-enhanced CT was used to distinguish between patients with and those without PAH by contrast material bolus propagation time and speed in the pulmonary arteries.^45^ Time differences between bolus peaks correlated with mean pulmonary artery pressures, and discrimination could be achieved with a sensitivity of 100% and specificity of 100% in patients without PH and a sensitivity of 93% and specificity of 80% in patients with PAH, respectively (Table 2).^45^

**Figure 1:**
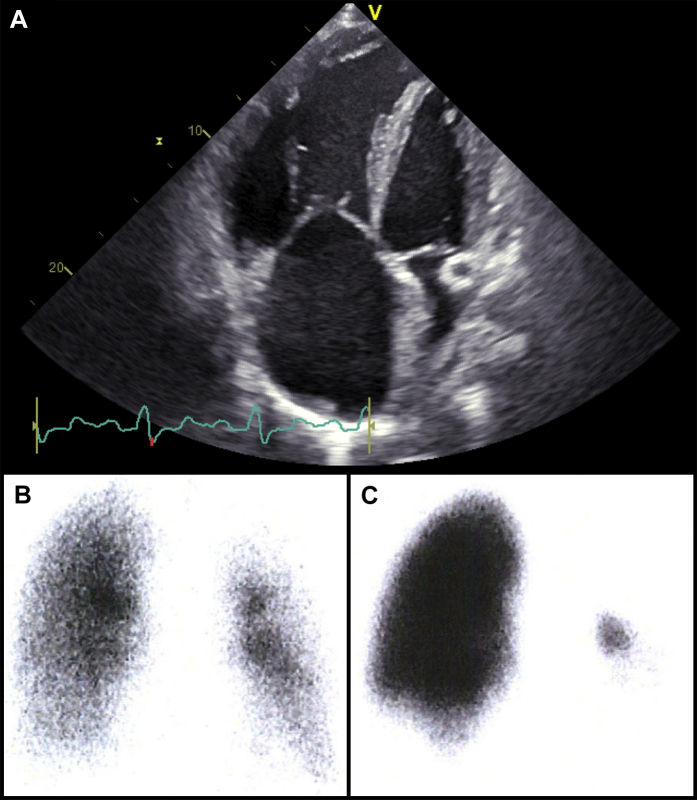
Imaging in a 24-year-old woman with a history of progressive shortness of breath on exertion, deep vein thrombosis, and recent hemoptysis. (**A**) Transthoracic echocardiographic 4-chamber view with severe right ventricular dilatation. (**B**) Technetium-99m–labeled aerosol ventilation and (**C**) perfusion images show nonmatched perfusion defects of right lower lobe and almost the entire left lung.

Suspicion should be high when the patient presents with a history of previous venous thromboembolism (VTE). Although formal screening cannot be recommended, CTEPH should be ruled out in any survivor of a pulmonary embolism with persistent dyspnea and > 15% perfusion defects 6 months after the acute VTE after at least 3 months of effective oral anticoagulation.^40^ V/Q planar images in at least 6 views combined with single-photon emission CT remains the preferred initial diagnostic test for CTEPH. CT pulmonary angiography (CTPA) has a sensitivity of detecting CTEPH of 51%, compared with a > 96% sensitivity of V/Q scanning.^62^ A normal V/Q, but not a normal CTPA, can exclude CTEPH, although scans tend to normalize as disease progresses.^63^ CTEPH may be the single PH subset in which advanced imaging and not RHC may be the primary approach to diagnosis, follow-up, and management. In the example shown in Figure 1, the correct diagnosis of CTEPH was made after an echocardiogram and a V/Q scan had been obtained. A CTEPH diagnosis was later confirmed by RHC and pulmonary angiography.

### Advanced imaging tools are useful for follow-up and management

An important more recent finding is that although PH is a pulmonary vascular disorder, structural and functional assessments of the right ventricle play a central role in both diagnosis and serial follow-up of patients with PAH.^23^ Therefore, it is reasonable that current guidelines suggest an integrated diagnostic algorithm in which noninvasive modalities are targeted to RV function and can be serially assessed to detect changes (Table 3); such an algorithm will play an ever more important role in the near future.^1^ For example, the use of 3D speckle tracking to assess area strain, radial strain, longitudinal strain, and circumferential strain correlates with clinical outcomes, with area strain and circumferential strain correlating best with RV ejection fraction.^70^ Stroke volume and RV ejection fraction measured by cMRI are the most commonly used parameters to evaluate global systolic RV function and to assess response to therapy.^74,75,79^ However, these parameters are highly dependent on preload and afterload and do not reflect RV contractility.^80^ RV end-systolic elastance (Ees) is accepted as a load-independent measure of intrinsic myocardial contractility. E_es_ is usually derived from pressure-volume loops by invasive conductance catheterization. Using this method, arterial elastance (E_a_) as a measure of RV afterload can also be determined. RV-to–pulmonary vascular (RV-PV) coupling, the adaptation of the right ventricle to its afterload, can be calculated by E_es_ divided by E_a_ (E_es_/E_a_ ratio). However, this method requires the assessment of pressure-volume loops during preload reduction by temporary balloon occlusion of the inferior vena cava, thus making it very invasive and potentially dangerous. As an alternative, E_es_/E_a_ can also be determined by combining measurements from standard RHC and MRI. Studies in healthy individuals and patients with PH have shown good agreement of MRI conductance catheterization data.^81,82^ The ratio of stroke volume–to–end-systolic volume (SV/ESV) that can be derived completely noninvasively from cMRI was found to correlate well with RV-PV coupling and to be a strong predictor of prognosis.^78^

## Conclusions

Because of the intrinsic properties of invasive diagnostics, the desire of patients, patient advocates, and physicians is that advanced imaging tools rather than hemodynamics will eventually become the primary approach to diagnosing, following, and managing PH. The values of sensitivities and specificities of available methods shown in Tables 1-3 allow for the selection of the best noninvasive tests for screening, diagnosis, and follow-up in PH, according to testing priorities. Although noninvasive assessment cannot currently replace RHC, it has become an essential part of the management paradigm for PH, and hopefully with further development will 1 day make RHC a historical curiosity.

## Funding Sources

This study was supported by FWF KLI209 and FWF F54 and by educational grants from Bayer (Grant No. 15662 [to C.G.]) and United Therapeutics Corporation (Grant No. REG-NC-002 [to M.G.]).

## Disclosures

I.M.L. has relationships with AOP Orphan Pharmaceuticals, Actelion, Bayer-Schering, Astra-Zeneca, Servier, Cordis, Medtronic, GSK, Novartis, Pfizer, and United Therapeutics. In addition to being investigator in trials involving these companies, relationships include consultancy service, research grants, and membership on scientific advisory boards. The other authors have no conflicts of interest to disclose.

## Figures and Tables

**Table 1:** Noninvasive imaging to screen for PH

First author	Technique	Number of patients	Study population/cause	Functional parameter/variable	Screening for PH	
					Sensitivity (95% CI), %	Specificity (95% CI), %
Denton et al.^24^	TTE	33	CTD (SSc)	sPAP	90	75
Parent et al.^25^	TTE	385	Sickle cell disease	Tricuspid regurgitation jet velocity	100	80
Rajaram et al.^26^	TTE	81	CTD	Tricuspid gradient	86	82
Wang et al.^27^	TTE	123	CHD	sPAP	89	84
Kuriyama et al.^28^	CT	23	Suspected PH	MPAD	69	100
Perez-Enguix et al.^29^	CT	71	Candidates for LTX	MPAD	66	86
Rajaram et al.^26^	CT	81	CTD	Ventricular mass index	85	82
Stevens et al.^30^	MRI	100	Suspected PH	PVR	92.5	85.2
Rajaram et al.^26^	MRI	81	CTD	RV mass index	85	82

CHD, congenital heart disease; CT, computed tomography; CTD, connective tissue disease; LTX, lung transplantation; MPAD, main pulmonary artery diameter; MRI, magnetic resonance imaging; PH, pulmonary hypertension; PVR, pulmonary vascular resistance; RV, right ventricular; sPAP, systolic pulmonary artery pressure; SSc, systemic sclerosis/scleroderma; TTE, transthoracic echocardiography.

**Table 2:** Noninvasive imaging to diagnose PH

First author	Technique	No. of patients	Study population/cause	Functional parameter/variable	Diagnosing PAH	
					Sensitivity (95% CI), %	Specificity (95% CI), %
Isobe et al.^46^	TTE	77	Controls vs suspected PH	RV acceleration time	93	97
Tei et al.^47^	TTE	63	Controls vs iPAH	Tei index	–	–
Saba et al.^48^	TTE	26	Suspected PH	sPAP	89	57
Hsu et al.^49^	TTE	49	CTD (SSc)	sPAP	58	96
Dahiya et al.^50^	TTE	26	Suspected PH	Corrected PVR Echocardiographic PVR	91 93	90 91
D'Alto et al.^51^	TTE	161	Suspected PH	Left atrial pressure Cardiac output mPAP PVR	85 – – –	– – – –
Gladue et al.^52^	TTE	248	CTD (SSc)	sPAP	94∗	73∗
Tan et al.^53^	CT	45	Suspected PH	MPAD	87	89
Chan et al.^54^	CT	101	Suspected PH	MPAD MPAD/AA ratio MPAD/DA ratio Right descending PA diameter RV/LV lumen ratio RV/LV wall ratio RV free wall True left descending PA diameter True right descending PA diameter	77 74 77 83 86 79 81 79 83	90 92 90 85 86 84 92 92 88
Corson et al.^55^	CT	305	Suspected PH	Right PA diameter MPAD	89 (85-94) 89 (84-93)	82 (74-89) 83 (76-90)
Helmberger et al.^56^	CT	24	Controls vs PH	Pulmonary vessel tortuosity	83	83
Pienn et al.^45^	CT	21	Controls vs PAH	Propagation contrast medium speed	100 (77-100)	100 (48-100)
Bouchard et al.^57^	MRI	27	Controls vs PAH	Left descending PA/DA MPAD MPAD/AA RV wall thickness Septal wall thickness	–	–
Saba et al.^48^	MRI	26	Suspected PH	Ventricular mass index	84	71
Sanz et al.^58^	MRI	59	Controls vs PAH	Average blood velocity Minimum PA area	93 (81-98) 93 (81-98)	82 (57-96) 88 (64-98)
Sanz et al.^58^	MRI	72	PH	Delayed contrast enhancement	–	–
Hsu et al.^49^	MRI	49	CTD (SSc)	MPAD	68	71
Nogami et al.^59^	MRI	20	Suspected PH	sPAP Stroke volume	– –	– –
Shehata et al.^60^	MRI	48	Controls vs PAH	RV longitudinal strain RV circumferential strain RV tangential strain	– – –	– – –
Swift et al.^61^	MRI	64	Suspected PH	sPAP PVR	87	90

AA, ascending aorta; CT, computed tomography; DA, descending aorta; CTD, connective tissue disease; iPAH, idiopathic pulmonary arterial hypertension; LV, left ventricular; MPAD, main PA diameter; mPAP, mean PA pressure; MRI, magnetic resonance imaging; PA, pulmonary artery; PAH, pulmonary arterial hypertension; PH, pulmonary hypertension; PVR, pulmonary vascular resistance; RV, right ventricular; sPAP, systolic PA pressure; SSc, systemic sclerosis/scleroderma; TTE, transthoracic echocardiography.

∗ In combination with pulmonary function tests.

**Table 3:** Noninvasive imaging to follow-up/detect change in PH

First author	Technique	No. of patients	Study population/cause	Functional parameter/variable	Detecting change in PAH/CTEPH/PH	
					Sensitivity (95% CI), %	Specificity (95% CI), %
Chow et al.^64^	TTE	28	Operable CTEPH before vs after PEA	Acceleration time Tricuspid regurgitation jet velocity	– –	– –
Eysmann et al.^65^	TTE	26	iPAH	Pericardial effusion Tricuspid early flow deceleration Pulmonary acceleration time	– – –	– – –
Tei et al.^47^	TTE	63	Controls vs iPAH	Tei index	–	–
Yeo et al.^66^	TTE	53	iPAH	Tei index	–	–
Raymond et al.^19^	TTE	81	iPAH	Right atrial area index Diastolic eccentricity index Pericardial effusion	– – –	– – –
Forfia et al.^67^	TTE	63	PAH	TAPSE	–	–
Dahiya et al.^50^	TTE	10	PAH	Corrected PVR Echocardiographic PVR	– –	– –
Fine et al.^68^	TTE	575	PH	RV longitudinal strain TAPSE	79 61	– –
Grünig et al.^69^	TTE	124	PAH/CTEPH	sPAP; response to exercise	77	53
Smith et al.^70^	TTE	97	PH	RV ejection fraction TAPSE RV area strain RV circumferential strain RV longitudinal strain RV radial strain	65 70 80 65 90 75	59 59 54 73 52 51
Courand et al.^71^	TTE	100	PAH	RV ejection fraction	–	–
Moledina et al.^35^	CT	31	Pediatric PAH	Fractal dimension	–	–
Zylkowska et al.^72^	CT	264	PAH/CTEPH	MPAD	95	39
van Wolferen et al.^73^	MRI	64	PAH	RV ejection fraction RV end-diastolic volume index LV end-diastolic volume index Stroke volume index	– – – –	– – – –
van de Veerdonk et al.^74^	MRI	76	PAH	RV ejection fraction	82	75
Freed et al.^75^	MRI	58	PH	RV ejection fraction RVIP-LGE	100	–
Ley et al.^76^	MRI	20	PAH/CTEPH	Cardiac output	–	–
Pandya et al.^77^	MRI	50	Pediatric PAH (CHD)	Septal curvature	83 (36-99)	91 (77-97)

CT, computed tomography; CHD, congenital heart disease; CTEPH, chronic thromboembolic pulmonary hypertension; iPAH, idiopathic pulmonary arterial hypertension; LGE, late gadolinium enhancement; LV, left ventricular; MPAD, mean PA diameter; MRI, magnetic resonance imaging; PA, pulmonary artery; PAH, pulmonary arterial hypertension; PEA, pulmonary thromboendarterectomy; PH, pulmonary hypertension; PVR, pulmonary vascular resistance; RV, right ventricular; RVIP, RV insertion point; sPAP, systolic PA pressure; SV/ESV, stroke volume/end-systolic volume ratio; TAPSE, tricuspid annular plane excursion; TTE, transthoracic Doppler echocardiography.
